# Genotype-Phenotype Correlations for Pathogenic *COL4A3–COL4A5* Variants in X-Linked, Autosomal Recessive, and Autosomal Dominant Alport Syndrome

**DOI:** 10.3389/fmed.2022.865034

**Published:** 2022-05-06

**Authors:** Judy Savige, Mary Huang, Marina Shenelli Croos Dabrera, Krushnam Shukla, Joel Gibson

**Affiliations:** Department of Medicine (Melbourne Health and Northern Health), Royal Melbourne Hospital, The University of Melbourne, Parkville, VIC, Australia

**Keywords:** genotype-phenotype correlation, Alport syndrome, *COL4A3*, *COL4A4*, *COL4A5*, XL Alport syndrome, AR Alport syndrome, AD Alport syndrome

## Abstract

Alport syndrome is inherited as an X-linked (XL), autosomal recessive (AR), or autosomal dominant (AD) disease, where pathogenic *COL4A3 – COL4A5* variants affect the basement membrane collagen IV α3α4α5 network. About 50% of pathogenic variants in each gene (major rearrangements and large deletions in 15%, truncating variants in 20%, splicing changes in 15%) are associated with “severe” disease with earlier onset kidney failure, and hearing loss and ocular abnormalities in males with XL inheritance and in males and females with AR disease. Severe variants are also associated with early proteinuria which is itself a risk factor for kidney failure. The other half of pathogenic variants are missense changes which are mainly Gly substitutions. These are generally associated with later onset kidney failure, hearing loss, and less often with major ocular abnormalities. Further determinants of severity for missense variants for XL disease in males, and in AD disease, include Gly versus non-Gly substitutions; increased distance from a non-collagenous interruption or terminus; and Gly substitutions with a more (Arg, Glu, Asp, Val, and Trp) or less disruptive (Ala, Ser, and Cys) residue. Understanding genotype-phenotype correlations in Alport syndrome is important because they help predict the likely age at kidney failure, and the need for early and aggressive management with renin-angiotensin system blockade and other therapies. Genotype-phenotype correlations also help standardize patients with Alport syndrome undergoing trials of clinical treatment. It is unclear whether severe variants predispose more often to kidney cysts or coincidental IgA glomerulonephritis which are recognized increasingly in *COL4A3-, COL4A4 -* and *COL4A5*-associated disease.

## Introduction

Alport syndrome is the commonest inherited kidney disease and the second commonest cause of inherited kidney failure after polycystic kidney disease (1). It is characterized by persistent haematuria and a family history of haematuria or kidney failure (2). The typical clinical features are seen in males with X-linked (XL) disease due to pathogenic *COL4A5* variants and in males and females with autosomal recessive (AR) disease and two pathogenic variants in *COL4A3* or *COL4A4*. These result in haematuria, kidney failure, hearing loss and ocular abnormalities. Affected basement membranes including the glomerular membrane (GBM) are thinned, lamellated, and moth-eaten. Autosomal dominant (AD) Alport syndrome is caused by a heterozygous pathogenic variant in *COL4A3* or *COL4A4* and associated with isolated haematuria and a thinned rather than lamellated membrane (3, 4). With AD Alport syndrome the risk of kidney failure is small, and hearing loss and ocular abnormalities are rare (5). AD Alport syndrome is also sometimes known as thin basement membrane nephropathy and represents the carrier state for autosomal recessive Alport syndrome (3).

## Structure of the Collagen IV Network

The six *COL4A1–COL4A6* genes code for the collagen IV α1–α6 chains (6). These genes belong to two families, *COL4A1*-like (*COL4A1, COL4A3*, and *COL4A5*) and *COL4A2*-like (*COL4A2, COL4A4*, and *COL4A6*) which have all arisen from reduplication of *COL4A1*. These six genes are highly homologous and the corresponding collagen IV α chains resemble each other structurally.

**Figure 1:**
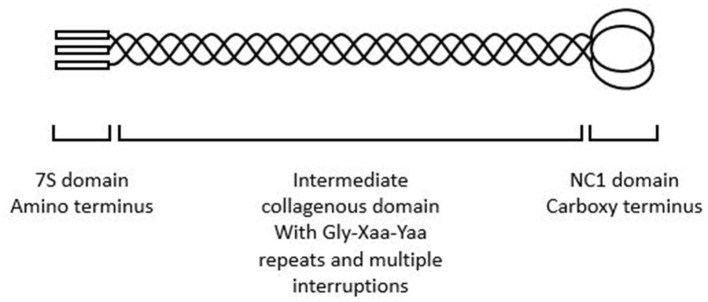
Collagen IV α3α4α5 heterotrimer demonstrating amino terminus, intermediate collagenous domain, and carboxy terminus. The intermediate collagenous domain comprises Gly-Xaa-Yaa repeats with multiple interruptions.

Each collagen IV α chain comprises a non-collagenous amino and carboxy terminus and an intermediate collagenous sequence with Gly-Xaa-Yaa repeats (Figure 1) as well as multiple interruptions that confer flexibility (6). All collagen chains include Gly-Xaa-Yaa repeats but the collagen IV α chains differ in that they retain the non-collagenous amino and carboxy termini through which they interact to form the α1α1α2, α3α4α 5, or α5α5α6 heterotrimers and the chicken wire networks that predominate in membranes.

The α1α1α2 network is widely distributed in basement membranes in infancy and is found in adults in the blood vessels, nerves and muscles (6). The α3α4α5 trimer is the major constituent of membranes in the glomerulus (GBM), cochlea and eye having replaced the α1α1α2 trimer at about 2 years of age. The α5α5α6 trimer is found in Bowman's capsule in the kidney and in skin.

To date, more than 5,000 pathogenic variants have been described in the *COL4A3–COL4A5* genes in the two main variant databases LOVD (https://www.lovd.nl/) and Clinvar (https://www.ncbi.nlm.nih.gov/clinvar/). These are both open access databases that accept submissions from diagnostic laboratories and from the literature. LOVD also includes clinical features for many variants which allows genotype-phenotype correlations. Many more variants have been reported for *COL4A5* than for *COL4A3* and *COL4A4* because genetic testing has been performed more often for individuals suspected of having X-linked Alport syndrome. Although the definitions of variant types differ in different databases, in general, pathogenic variants in each of the *COL4A3–COL4A5* genes include 15% major rearrangements or large deletions, 20% truncating variants, 15% splicing changes, and 45% missense variants (Table 1).

**Table 1:** Types of all pathogenic variants in *COL4A5, COL4A3*, or *COL4A4*.

	**Large rearrangements/deletions/indels**	**Truncating variants (nonsense and frameshift)**	**Splice site**	**Missense**
**LOVD**				
*COL4A5* (*n* = 1,967)	20%	23%	14%	49%
*COL4A3* (*n* = 383)	16%	21%	6%	60%
*COL4A4* (*n* = 335)	21%	35%	8%	44%
**ClinVar**				
*COL4A5* (*n* = 1,031)	99 (10%)	321 (31%)	137 (13%)	514 (50%)
*COL4A3* (*n* = 259)	27(10%)	106 (41%)	44 (17%)	82 (32%)
*COL4A4* (*n* = 249)	23 (9%)	116 (47%)	41 (16%)	69 (28%)

*Definitions of large rearrangements etc. differ for LOVD and ClinVar. https://databases.lovd.nl/shared/genes/COL4A5/graphs; https://simple-clinvar.broadinstitute.org/ filtered for Pathogenic and Likely pathogenic, expressed as % of all Pathogenic and Likely Pathogenic variants found. The numbers include occasional variants twice because of features in overlapping categories*.

“Severe” variants in *COL4A5* are more likely to be rearrangements or deletions, truncating, or splicing changes than missense variants (7). This means that overall severe changes account for about 50% of all pathogenic variants in each of these genes, and thus that the likelihood of finding a severe variant in a person with Alport syndrome is currently also 50%.

Deletions, frameshift variants, and termination codons all result in loss of the corresponding α chain from nonsense-mediated decay and subsequently of the collagen IV α3α4α5 triple helix from affected membranes. This is replaced with the α1α1α2 network (8) which is more susceptible to proteolysis (9). Initially the GBM is thinned but repeated episodes of damage and repair result in a lamellated, moth-eaten appearance. Splicing variants have different effects depending on whether they produce a truncating change or exon skipping (10).

In contrast, the α chains resulting from a missense variant are often associated with a disrupted trimer that is retained within the podocyte ER (11, 12), increasing ER stress and podocyte loss (13, 14). Of all the missense variants, the majority (30%) are Gly substitutions in the intermediate collagenous domain. Gly is the smallest amino acid that fits within the interior of the triple helix but can be replaced by 8 different amino acids or a termination codon (7). Most Gly substitutions are pathogenic because substitution with a larger amino acid distorts triple helix formation.

## Advantages of Predicting Severity of Pathogenic Variants

It is useful for the clinician to be able to predict whether a pathogenic *COL4A3–COL4A5* variant is likely associated with severe or mild features in Alport syndrome. The typical severe form is seen in males with X-linked disease who have recurrent macroscopic haematuria, early onset proteinuria and kidney failure, that generally requires dialysis or a kidney transplant well before the age of 30 years (15–17). The GBM may be thinned or lamellated, with the collagen IV α5 chain absent from a kidney or skin biopsy. Lenticonus, central fleck retinopathy, and temporal retinal atrophy are common, and a maculopathy or macular hole may occur (18, 19). In contrast “mild” disease is associated with haematuria, later onset proteinuria and kidney failure often in middle age, together with a thinned, possibly partly lamellated, GBM (20). There may be a late onset hearing loss, and a peripheral retinopathy is common (21).

The severe and mild phenotypes are subtly different in other forms of inheritance. Females with a severe pathogenic *COL4A5* variant have an increased risk of proteinuria, and hearing loss is common but lenticonus does not occur (22). With AR Alport syndrome, two severe variants in *COL4A3* or *COL4A4* have a more damaging effect than one or none (23). The relationship is less clear for severe heterozygous *COL4A3* or *COL4A4* variants in AD Alport syndrome since kidney failure is not common and may be due to coincidental disease.

The advantages of being able to predict severe variants include being able to institute treatment to delay kidney failure onset. Renin-angiotensin-aldosterone system blockade delays kidney failure for years in males with X-linked disease, and the delay may be sufficiently long in women that they do not require dialysis or a kidney transplant (24, 25). SGLT2 inhibitors have demonstrated promise in Alport syndrome and are currently undergoing further investigation (26, 27). Severe variants may also indicate the need for earlier, and more aggressive, or more expensive interventions as they become available (28, 29). Some treatments are specific for severe disease. Demonstrating a severe variant may also represent a persuasive argument for dissuading an affected family member from acting as a kidney donor. In addition, recognizing severe variants is important in clinical trials where standardizing the risk of kidney failure helps compare treatment outcomes.

## X-Linked Alport Syndrome in Males

The location where a pathogenic variant affects the collagen α chain (amino terminus, intermediate collagenous domain, interruptions, or carboxy terminus) and the variant type (large rearrangements, truncating, splicing, or missense variants) are the major determinants of variant severity and age at kidney failure, and likelihood of hearing loss and ocular abnormalities. Because pathogenic *COL4A5* variants result in kidney failure in most males the genotype-phenotype relationship is understood best in males with X-linked disease.

Many studies of *COL4A5* variants in males have found that severe changes (large rearrangements, nonsense and frameshift, and splicing variants) are associated with an earlier age at kidney failure than missense changes (15–17, 30). Splice site variants often have an effect intermediate between truncating and missense variants (16).

In the *COL4A5* variants in the LOVD database, the mean age at kidney failure for all males was 25.1 ± 10.6 years (*n* = 326). Some studies have suggested that pathogenic changes in the first 20 exons of *COL4A5* and hence the amino terminus of the collagen IV α5 chain is associated with milder disease (17) and others have found more severe disease with earlier onset kidney failure (16). In LOVD, the average age at kidney failure for variants in the amino terminus was 20.3 ± 5.4 (*n* = 14), 25.6 ± 11.1 years for variants in the intermediate domain (*n* = 262) and 24.0 ± 8.2 (*n* = 50) for those in the carboxy terminus suggesting a more severe effect for the amino terminus compared with the intermediate domain (*p* = 0.08) but not the carboxy terminus (*p* = 0.12). However 11 of the 14 amino terminus changes were deletions which have a more severe outcome and were likely to have biased the results. Thus variant types must also be taken into account before assessing the severity associated with a variant location.

In the LOVD database, the average age at kidney failure for men with deletions was 21.1 ± 6.8 years (*n* = 75) which was earlier than the overall mean age (*p* = 0.002) and for those with nonsense variants was 20.4 ± 5.0 years (*n* = 33, *p* = 0.01). The mean age for men with canonical splice site variants was 25.2 ± 10.7 years (*n* = 45, *p* = 0.95). Donor splice site changes have been associated with earlier onset kidney failure than acceptor variants (17) but the mean age for splice site variants at −1 or −2 was 25.2 ± 11.6 years (*n* = 21) and 25.1 ± 10.2 (*n* = 24) for variants at +1 or + 2 (*p* = 0.98 compared with each other, and *p* = 0.95 compared with the overall mean). Duplications were associated with a mean age of 23.9 ± 6.1 years (*n* = 15, *p* = 0.66 compared with overall mean).

In the LOVD database, Gly substitutions in the intermediate collagenous domain [mean age at kidney failure of 28.4 ± 12.4 years (*n* = 52)] had a worse outcome than non-Gly substitutions [40.7 ± 17.6 years (*n* = 5), *p* = 0.03] (16).

Gly substitutions represent the commonest missense variants in *COL4A5*, currently accounting for most unique pathogenic variants in males (124/147, 84%).

Gly substitutions distant from a non-collagenous sequence (interruption, amino, or carboxy terminus) had a more severe phenotype with earlier onset kidney failure than Gly substitutions adjacent to a non-collagenous sequence (31). In addition substitutions with highly destabilizing residues such as Glu, Asp, Arg, Val, or Trp were associated with earlier onset kidney failure than substitutions with the less-destabilizing, Ala, Ser, or Cys (31). In LOVD, the mean age at kidney failure was 24.6 ± 8.6 for severe Gly substitutions (*n* = 83) and 31.1 ± 14.2 for non-severe substitutions (*n* = 68, *p* = 0.0007).

Treatment also affects the age at kidney failure for truncating variants and non-truncating variants differently. The age at kidney failure for both truncating and non-truncating variants is delayed by RAAS blockade but treatment delayed kidney failure more for non- truncating variants (30).

Severe variants are also associated with hearing loss (15–17), and ocular abnormalities, in particular, lenticonus, and fleck retinopathy (15–17). No effect of variant severity has been demonstrated on macroscopic haematuria, hypertension, or GBM thickness (15, 16).

## X-Linked Alport Syndrome in Females

The correlation between *COL4A5* variant location, type and phenotype has been less clear for females with X-linked Alport syndrome. Indeed an early large study found no genotype-phenotype correlation in women with X-linked disease (32). They also found no correlation between earlier age at kidney failure in men and women from the same family (32).

Part of the lack of correlation in women has been attributed to random X chromosome inactivation (33). However it is also likely to be partly due to so few women developing kidney failure and, of those who do, this occurring in later life. Women develop hearing loss and ocular abnormalities less often too. Thus recent studies have focused instead on the relationship between genotype and proteinuria since proteinuria typically precedes kidney failure (34).

Thus a recent large study of 336 females with genetically-proven X-linked Alport syndrome from 179 families where about half the pathogenic variants were severe found that 175 (73%) had proteinuria at a median age of 7 years (34). Fifty-two of the 336 (15%) had kidney failure by the age of 65 years.

Another smaller cohort found that severe variants (all those other than missense changes) were more commonly associated with proteinuria and impaired kidney function than missense variants (35). While this cohort also included occasional individuals with digenic inheritance these were unlikely to have had a significant effect.

No studies in women with pathogenic *COL4A5* variants have demonstrated any genotype-phenotype correlations for hearing loss or ocular abnormalities.

In summary there may be a similar genotype-phenotype correlation in women with pathogenic *COL4A5* variants and proteinuria as occurs in men with kidney failure but with a smaller effect that requires a larger cohort for its demonstration. Other factors contributing to the age at kidney failure as well as X chromosome activation, include coincidental causes including hypertension, diabetes and obesity.

## AR Alport Syndrome

Individuals with AR Alport syndrome have two pathogenic variants in *COL4A3* or *COL4A4*, and the *COL4A3* and *COL4A4* genes are affected equally often. In the LOVD database, the age at kidney failure was not different for AR and for X-linked Alport syndrome (24.4 ± 7.8 years, *n* = 237, *p* = 0.39), and in the case of AR disease, was not different for *COL4A3* (23.2 ± 9.3, *n* = 35, *p* = 0.45) or *COL4A4* (25.4 ± 10.3, *n* = 26, *p* = 0.55) variants (7).

Individuals with two truncating variants were more likely to develop kidney failure before 30 years of age than those with only one truncating variant, who were in turn more likely than those with no truncating variant (23). This correlation has now been confirmed in a small but unrelated cohort where both age at kidney failure and age at hearing loss were earlier with at least one truncating variant than with none (36). A further study confirmed that the presence of at least one milder (missense) variant delayed kidney failure onset (*p* = 0.024), and that kidney survival was further increased with two missense variants [*p* = 0.016; (37)]. In this study, individuals with missense variants had a later onset of kidney failure, hearing loss and ocular abnormalities (37).

## AD Alport Syndrome

There are now several large studies and a meta-analysis of genotype-phenotype analyses in individuals with AD Alport syndrome (5, 31, 38, 39), but again the difficulty is that kidney failure is uncommon in these individuals, a large cohort might be needed to demonstrate a small effect, and kidney failure may result from coincidental causes. However again proteinuria may represent a surrogate marker for kidney failure (5).

A recent analysis of the UK 100K Genomes Project database demonstrated that certain variant types were associated with a higher penetrance of haematuria (31). It examined *COL4A3* and *COL4A4* variants that resulted in a Gly substitution and found that substitutions with a highly destabilizing residue (Arg, Glu, Asp, Val, and Trp) were associated with an increased risk of haematuria [*p* = 0.018; (31)], and that substitutions adjacent to a non-collagenous interruption or amino or carboxy terminus were associated less often with haematuria (*p* < 0.001). There was no association between haematuria and proximity to the amino terminus for missense variants that were Gly substitutions.

A further study of 240 individuals from 78 families with genetically-proven *COL4A3* or *COL4A4* variants, included 61 who developed kidney failure (24%) at a median of 67 years (58–73) (5). Fifty-seven percent of their variants were missense and the others were “severe.” In this study there was no difference in the age at kidney failure for truncating variants or for missense variants that were or were not Gly substitutions (*p* = 0.3); nor for Gly substitutions, splicing or missense variants (*p* = 0.90); nor Gly substitutions with Arg, Glu, or Asp or other severe non-missense variant types compared with other Gly substitutions [*p* = 0.5; (5)]. There was however a large intrafamilial variability in age at kidney failure.

A further study found no difference in the age at developing proteinuria or kidney failure for missense and non-missense variants (38).

However a meta-analysis of family members with 74 variants where 20 were not missense found the median age at kidney failure was 55 years for missense variants and 47 years for non-missense changes [*p* = 0.02; (39)].

At least two of these studies had a very wide variation in age at kidney failure (5, 39).

## Hypomorphic Variants

Hypomorphic or variants associated with milder disease are recognized increasingly in the *COL4A3–COL4A5* genes (Table 2). They are often clinically unrecognised and are commonly found in normal variant databases such as gnomAD (40). They may have features that are the opposite of severe variants such as occurring in women (for *COL4A5* changes); and for all *COL4A3–COL4A5* variants, missense variants, especially Gly substitution with Ala, Ser, or Cys; or Gly substitutions adjacent to a non-collagenous terminus or interruption; or non-Gly substitutions.

**Table 2:** Clinical phenotype of severe and hypomorphic variants.

**Mode of inheritance**	**Severe**	**Hypomorphic**
X-linked Alport syndrome in males	Episodes of macroscopic haematuria with intercurrent infections; persistent haematuria, proteinuria; early onset kidney failure, hearing loss; central fleck retinopathy; lamellated GBM; more severe FSGS	Haematuria, intermittent haematuria or none; less and later onset proteinuria and FSGS; late onset kidney failure; late onset hearing loss; no lenticonus, less central retinopathy; thinned GBM or patchy lamellation
X-linked Alport syndrome in females	Haematuria, proteinuria; possibly late onset kidney failure; more widespread lamellation; FSGS	Haematuria, intermittent haematuria or none; late onset proteinuria if at all
Autosomal recessive Alport syndrome	Haematuria, proteinuria; early onset kidney failure, hearing loss; central fleck retinopathy	One or two hypomorphic variants delays age at kidney failure
Autosomal dominant Alport syndrome	Haematuria, possibly proteinuria; FSGS	Haematuria, intermittent haematuria or none

*GBM, glomerular basement membrane; FSGS, focal and segmental glomerulosclerosis*.

Thus, two of the commonest hypomorphic variants are p.Gly624Asp in *COL4A5* and p.Leu1474Pro in *COL4A3*. p.Gly624Asp in *COL4A5* is found in about one in 3,000 of the European population or about one quarter of all Europeans with X-linked Alport syndrome (20, 41). This variant is located immediately adjacent to a non-collagenous interruption in the intermediate collagenous domain which contributes to its mild phenotype. It is associated with late onset kidney failure, hearing loss, but a normal ocular examination, and often a thinned rather than lamellated GBM.

The p.Leu1474Pro variant in *COL4A3* occurs in up to one in 200 people. The substitution occurs in the carboxy non-collagenous domain. On its own it may be associated with a normal urinary sediment, proteinuria and FSGS (42) or in association with another *COL4A3* variant, with AR Alport syndrome, and kidney failure.

## Predicting Severe Variants

In summary, the determinants for variant severity appear to be the same for all the *COL4A3–COL4A5* genes (Table 3). The renal physician may be assisted in deciding on the variant severity in their patient after consultation with a clinical geneticist and a multidisciplinary team. About half the pathogenic variants in each gene (major rearrangements, large deletions, truncating variants, some splicing variants) are associated with more severe disease, with earlier onset proteinuria and kidney failure, hearing loss, and ocular abnormalities in males with XL and in males and females with AR disease. For AD Alport syndrome severe changes may only increase the penetrance of haematuria. Interestingly a correlation between severe variants and proteinuria may be emerging for women with XL disease.

**Table 3:** Factors determining severity of genetic variants in the *COL4A3–COL4A5* genes.

**Variant feature**	**Detail**	**Severity**
Location	Amino terminus	Severity probably depends more on type than location
	Collagenous domain	
	Carboxy terminus	
Type	Rearrangements, large deletions	Severe
	Truncating variants	Severe
	Splice site variants	Severe if they result in truncation
	Missense variants	
Gly substitution	Gly substitutions	More severe generally than non-Gly substitutions
	Position 1 Gly substitutions	More severe than non-position 1 substitutions
	Position 1 Gly substitutions not adjacent to interruptions	More severe than variants adjacent to interruptions
	Gly substitutions with Arg, Asp, Glu, Trp, or Val	More severe than Gly substitutions with Ala, Ser, or Cys

*More severe variants are associated with a younger age at kidney failure or proteinuria*.

There are also two objective methods for predicting the clinical effects of *COL4A3–COL4A5* variants but these are not widely available. One is immunohistochemical staining for the collagen IV α5 chain in the kidney or skin (43). Males with absent staining have a more abnormal GBM and a worse prognosis than those with positive staining (44, 45). The other method is still a research tool but uses an *in vitro* expression system to examine collagen IV α3α4α5 heterotrimer formation and secretion (46). Reduced formation and secretion correlate with proteinuria development and early onset kidney failure (46).

## Caveats to Predicting Clinical Features From Genotype

However there are also caveats to predicting the clinical features from the genotype based on previously reported studies.

Firstly, there is a bias to reporting more severe disease. Most publications are of hospital—based series where affected individuals have the typical Alport features with kidney failure.

Secondly, sometimes the phenotype varies even in family members with the same pathogenic variant. Twenty-three families with XL Alport syndrome, each with at least 3 males with kidney failure, included 17 families (74%) where men had a consistent age at kidney failure and six with varying ages (26%) (15). Missense and splice site variants are especially associated with divergent ages. Other studies have found more consistency (15–17). Women with XL disease and males and females with AD Alport syndrome are the populations with the most variation in the age at kidney failure. This is possibly because they have a smaller genetic risk of kidney failure and their likelihood and age may be distorted by coincidental diseases such as hypertension, obesity, diabetes, or modifying variants in other filtration barrier genes (47).

Thirdly, the mode of inheritance must be considered. It has been more difficult to correlate *COL4A5* variants in women with age at kidney failure because of random X chromosome inactivation. Women and girls have heterozygous *COL4A5* variants twice as often as men but typically have milder disease (22). Only 20% have kidney failure by the age of 60 years but hearing loss and peripheral fleck retinopathy are common (32). Milder disease occurs because, overall with balanced X inactivation, only half the female podocytes are affected and produce defective trimers. When X inactivation is skewed, a higher or lower proportion of podocytes are affected, and the phenotype varies correspondingly. With autosomal recessive inheritance, the clinical severity depends on the consequences of both variants.

Fourthly, genetic testing techniques have changed over the past 20 years of reporting genotype-phenotype studies. Many families with typical disease underwent testing when it first became available and those tested now are often demonstrated to have milder variants (Table 4). In addition, the currently- used technique of Whole Exome Sequencing is less sensitive for detecting large deletions, and hypomorphic variants are recognized increasingly (48).

**Table 4:** Average age at kidney failure in males with pathogenic *COL4A5* variants in LOVD in 2021 compared with 2016.

	**Up to 2016** **(*n* = 1,168) (7)**	**2021 (*n* = 35)**	**OR (95%CI), *P*-value**
Missense	504 (43%)	20 (57%)	*OR* = 0.57 (0.29–1.12), *p* = 0.10
Non-missense (severe)	664 (57%)	15 (43%)	
Ave age at kidney failure	24.4 ± 7.8 years	29.2 ± 14.6 years	*p* = 0.0026

*Relatively more missense (non-severe) variants were detected in 2021 which was consistent with the later age at onset of kidney failure. This may be because families with very severe disease had already been identified*.

Fifthly, commonly-used treatments such as renin-angiotensin-aldosterone inhibitors modify the age at kidney failure in Alport syndrome (24, 25). Treatments may also modify the hearing loss and ocular abnormalities.

## Future Studies

Future studies will focus on confirming more closely a genotype-phenotype correlation using proteinuria rather than kidney failure in women with X-linked Alport syndrome and in individuals with heterozygous *COL4A3* or *COL4A4* pathogenic variants. It may be possible to develop a more precise algorithm for predicting the age at kidney failure. More information will be available about hypomorphic variants, and further studies may explain whether severe *COL4A3–COL4A5* variants predispose to IgA nephropathy and cystic kidney disease, or reduce the risk of diabetic nephropathy (49).

The detection of a severely-damaging genetic variant represents a powerful argument for starting treatment with ACE inhibitors, and possibly other more specific treatments much earlier in order to delay the onset of end-stage kidney failure.

## Author Contributions

JS designed the project and wrote the first draft. MH, MC, and KS undertook the analysis of all the variants in the LOVD database. JG read the manuscript, provided the figure, added to the discussion, and corrected the draft. All authors reviewed and approved the final manuscript.

## Conflict of Interest

The authors declare that the research was conducted in the absence of any commercial or financial relationships that could be construed as a potential conflict of interest.

## Publisher's Note

All claims expressed in this article are solely those of the authors and do not necessarily represent those of their affiliated organizations, or those of the publisher, the editors and the reviewers. Any product that may be evaluated in this article, or claim that may be made by its manufacturer, is not guaranteed or endorsed by the publisher.
